# A Phase-Change Mechanism of GST-SL Based Superlattices upon Sb Flipping

**DOI:** 10.3390/ma14020360

**Published:** 2021-01-13

**Authors:** Teng Sun, Furong Liu, Jicheng Guo, Gang Han, Yongzhi Zhang

**Affiliations:** 1 Key Laboratory of Trans-Scale Laser Manufacturing Technology, Beijing University of Technology, Ministry of Education, 100 Ping Leyuan, Chaoyang District, Beijing 100124, China; sunny@emails.bjut.edu.cn (T.S.); smart.guo@hotmail.com (J.G.); yeahhanpei@163.com (G.H.); ZhangYZ@bjut.edu.cn (Y.Z.); 2 Beijing Engineering Research Center of Laser Technology, Beijing University of Technology, 100 Ping Leyuan, Chaoyang District, Beijing 100124, China; 3 Institute of Laser Engineering, Faculty of Materials and Manufacturing, Beijing University of Technology, 100 Ping Leyuan, Chaoyang District, Beijing 100124, China

**Keywords:** superlattices, GST-SL, AIMD simulation, flipping, PCMs

## Abstract

Reversible phase-change behaviors of Ge–Sb–Te based superlattices (GST-SL) were studied by ab initio molecular dynamics (AIMD) simulations based on three models containing Ge/Sb intermixing, namely the Petrov-mix, Ferro-mix, and Kooi-mix models. The flipping behavior of Sb atoms was found in all the three GST-SL models in the melting process. Among them the Kooi-mix model exhibited the best stability, and the analyses of bond length distribution and electron localization function provided a better explanation on the phase transition of GST-SL. Finally, we proposed a fast switching model for GST-SL based on Sb flipping.

## 1. Introduction

The use of Chalcogenide materials-based data storage devices (PCM) instead of the current flash memory technology has been explored for unprecedented applications, such as the Internet of Things and neuromorphic computing [1]. Recently, GeTe/Sb_2_Te_3_ superlattice (GST-SL), a typical one of chalcogenide alloys, was found to own a better performance in speed and power dissipation than the general Ge_2_Sb_2_Te_5_ (GST) material [2]. Several studies have in addition identified some properties, such as topological insulating [3] and magnetoresistance [4], etc., in the GST-SL. Although an enormous potential for use in photoelectric functional material has been shown, the atomic structure and phase-change mechanism of GST-SL is still contentious [5].

The Ge atoms switching mechanism in GST-SL superlattice was firstly proposed by Simpson et al. [2]. Then, some atomic structure of GST-SL, including Kooi, Petrov, Inverted_Petrov and Ferro models, were summarized by Tominaga et al. [6]. Some phase transition models based on Ge atoms, such as Petrov to Inverted_Petrov transition, Ferro to Inverted_Petrov transition [7], and strain assisted transition (based on Petrov model) [8], were subsequently proposed. However, the recent studies [9,10] based on high resolution TEM images showed that Ge/Sb atom intermixing existed in the GST-SL, and the transition based on a Ge/Sb intermixing model was considered to be more reasonable than based on the previous models [11,12]. In this paper, we studied the phase change behavior of GST-SL with the consideration of Ge/Sb intermixing via the ab initio molecular dynamics (AIMD) and the mechanism of phase transition of GST-SL was then discussed.

## 2. Materials and Methods

The simulation was performed using the VASP codes [13]. Perdew–Burke–Ernzerhof generalized gradient approximation (GGA-PBE) method [14,15] was adopted with 180 eV energy cutoff, 3 fs time step, gamma point, 0.1 eV Gaussian smearing, NVT(constant number, volume, and temperature) ensemble, and periodic boundary conditions. In this AIMD simulation, we built three GST-SL models with Ge/Sb intermixing (named Petrov-mix, Ferro-mix, and Kooi-mix, respectively). The Ge:Sb mixing ratio of 25% for GST-SL models referred to the density functional theory (DFT) calculations [11] (the detailed structure can be seen from the Figure S1 of Supplementary Materials). The total number of atoms in each periodic model was 144, with the individual atom numbers of Ge = 36, Sb = 36, and Te = 72.

## 3. Results

**Figure 1:**
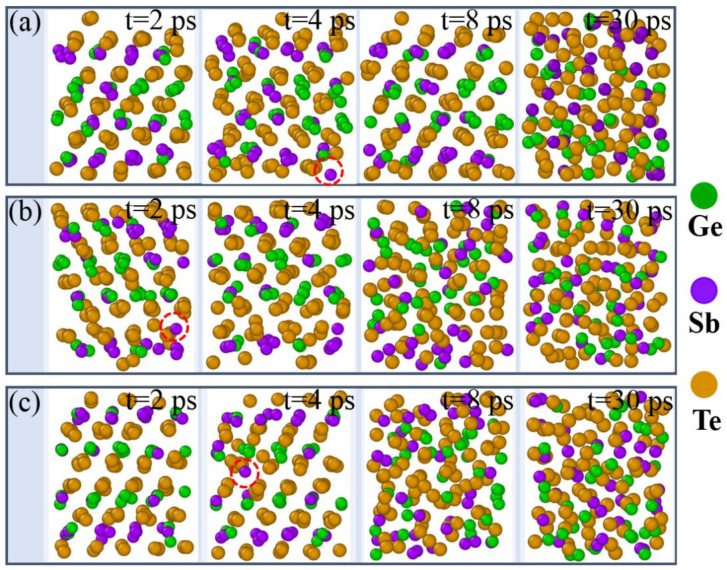
Snapshots of the melting process of GST-SL modes under 1400 K at different times (t = 2, 4, 8, 30 ps). (**a**–**c**) show the Kooi-mix, Ferro-mix, and Petrov-mix, respectively.

Generally, the amorphization (RESET) process of phase change materials is achieved by melting at a constant high temperature, and the melting process is usually accompanied by a rapid increase in free energy [16]. According to our heating simulation (see Figure S2 of Supplementary Materials), 1400 K was selected for the melting simulation of GST-SL model. The total time for the melting simulation was 35 ps. Figure 1 shows the snapshots of GST-SL models in the different times of 2, 4, 8, 30 during the amorphization process. According to the Figure 1a, we can see that the Kooi-mix model maintains a relatively order structure at the beginning of the melting process (t = 2 ps). Interestingly, a Sb atom (marked by the red circle) enters into the Te–Te Vander Waals layer (vdW gap) at 4 ps and returns to the original position at 8 ps. When time goes to 30 ps, the Kooi-mix model turns into a pure amorphous structure. We infer that there exists a metastable state between the crystal and pure amorphous states. Figure 1b displays the amorphization simulation of Ferro-mix model. The phenomenon that a Sb atom (in the red circle) gets to the Te-Te layer is also found at 2 ps. Interestingly, the Petrov-mix model also owns the same characteristic about the Sb atom in Figure 1c (t = 4 ps). Hence, the behavior of Sb atom entering into the vdW gap is a common characteristic of these GST-SL models. Besides, by comparing the snapshots of the three GST-SL models at 8 ps, we can see that Kooi-mix model is more stable than the other models obviously. In summary, the Sb atom of GST-SL is more inclined to enter into the vdW gap at elevated temperature than the Ge atoms, and there exists a metastable state between the crystal and amorphous states for GST-SL.

**Figure 2:**
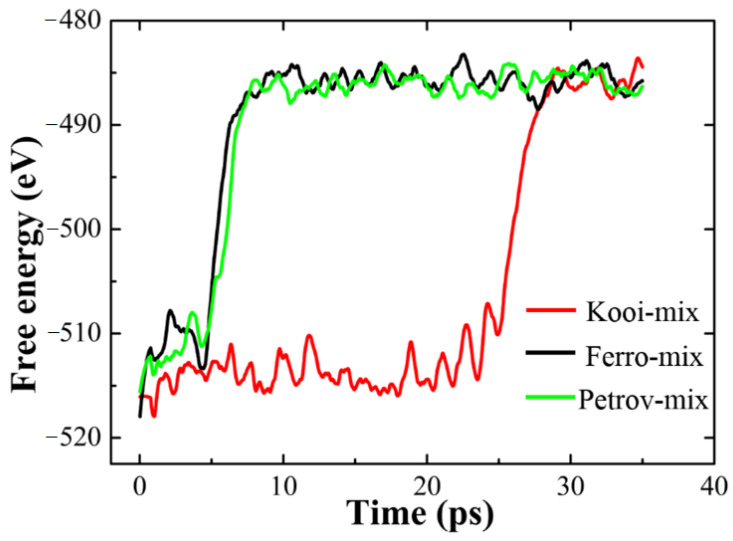
Free energy variations of the three GST-SL models during the melting process at 1400 K.

In the AIMD simulation, free energy of an atomic model will increase due to the absorption of latent heating. This property is usually employed to trace the melting process [16]. To compare the melting process of these GST-SL models well, we collected the information of free energies for GST-SL models during the melting process at 1400 K (shown in Figure 2). Obviously, the free energies of the three GST-SL models rise to the same level about −485 eV at 35 ps, which corresponds to the phenomenon that all the crystal GST-SL models convert into a pure amorphous state at the end of melting simulation in Figure 1. Besides, the time for order-to-disorder transition is only 2–3 ps for the three models according to the free energy curves. The difference is that melting time for both the Ferro and Petrov models is less than 8 ps, but that for Kooi-mix model is nearly 30 ps. This result once again proves that the Kooi-mix model is more stable. Given that the structure of RESET state for GST-SL is a partial amorphous state [2,5,8,17], the metastable state of GST-SL (a Sb atom enters into the vdW gap) is considered as the beginning of the RESET process of GST-SL.

**Figure 3:**
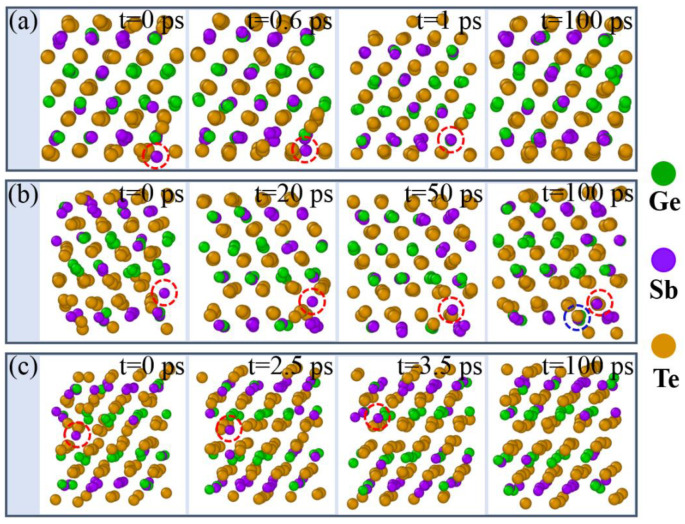
Key snapshots of GST-SL models at different times during the 700 K annealing, (**a**–**c**) show the RESET structures of the Kooi-mix, Ferro-mix, and Petrov-mix model, respectively.

In order to investigate crystallization properties of GST-SL, we performed the annealing simulation at 700 K for 100 ps based on RESET structures obtained in the melting process. Figure 3a shows key snapshots of a Kooi-mix RESET structure during the annealing process. It can be seen that a Sb atom (marked by red circle) locates in the vdW gap initially, and begins to cross the Te layer at 0.6 ps. When the time goes to 1 ps, this Sb atom returns to the Ge/Sb layer and the overall structure of Kooi-mix model recovers to the previous crystalline state (SET state). Additionally, this crystalline structure exhibits an excellent stability on the 700 K condition according to the snapshot at 100 ps. Figure 3b displays the annealing process of the Ferro-mix RESET model. Obviously, the Sb atom at the vdW gap in the Ferro-mix model is unable to come back to the previous position (Ge/Sb layer) as like that in the Kooi-mix model. This is because the way to Ge/Sb layer is blocked by a Te atom (marked by blue circle). In the Petrov-mix model simulation (Figure 3c), the Sb atom returns to the Ge/Sb layer from the vdW gap at 3.5 ps. Similarly, this crystalline structure of the Petrov-mix was also stable in the 100 ps annealing simulation. On the basis of these results, we conclude that the Sb atoms in the vdW gap of GST-SL models have the tendency to return to the previous Ge/Sb layer in the annealing condition, and this transition can be completed in several picoseconds.

**Figure 4:**
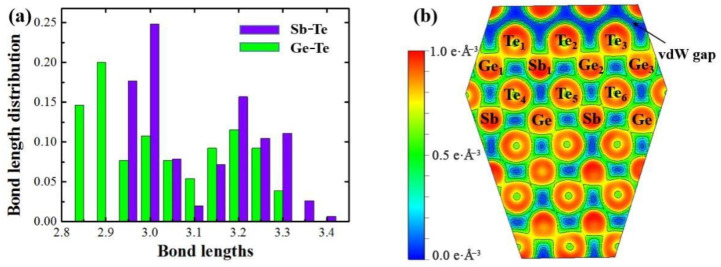
(**a**) Bond length distribution and (**b**) electron localization function (ELF) of the crystalline Kooi-mix model. The color scale for the ELF value is given at the left of the Figure 4b.

According to the melting and crystallization simulation above, we can see that Kooi-mix model exhibited the best performance in stability and crystallization velocity. Hence, the Kooi-mix model is considered helpful to reveal the phase-change mechanism of GST-SL. Figure 4a shows the bond length distribution of the Kooi-mix model in the crystalline (SET) state. It is obvious that the Ge–Te and Sb–Te bonds are in the range of 2.83–3.31 Å and 2.93–3.39 Å, respectively. This distribution is similar to that in crystalline Ge_2_Sb_2_Te_5_ alloy [17,18]. Besides, the lengths of Ge–Te bonds are mainly in the range of 2.85–2.90 Å, but Sb–Te bonds are mainly in 2.95–3.0 Å. Therefore, Ge–Te bonds are considered to be shorter and more stable than Sb–Te bonds in the Kooi-mix model. Hence, Ge–Te bonds have stronger impact on the structure stability of GST-SL than Sb–Te bonds. However, the maximum length of Sb–Te bonds (3.39 Å) is bigger than that in Ge–Te bonds (3.31 Å), so the impact of Sb–Te bonds is farther in the distance. According to our calculation, average distances of Ge–Te, Sb–Te, and Te–Te bonds are 3.04, 3.11, and 4.31 Å, respectively. That is to say, the Te-Te van der Waals interface (vdW gap) is the weakest connection in GST-SL. Therefore, the phase-change behavior of GST-SL is more likely to occur in the position of vdW gap when heated. Generally, the whole structure between the vdW gaps is considered as a block [9,10,19]. As shown in Figure 1a, the block of the Kooi-mix model owns a sequence: Te–Te–Sbrich–Te–Gerich–Te–Gerich–Te–Sbrich–Te–Te. Obviously, owning the characteristics that the Sb-rich layer is near the vdW gap, and the Ge-rich layer is the inner part of the block. For this reason, stability of GST-SL should be dominated by the strong impact of Ge-Te bonds, which can be employed to explain why the Kooi-mix mode is more stable than the other two models above.

Figure 4b exhibits the electron localization function [20] (ELF) of the crystalline Kooi-mix model. It can be seen that there is no electron in the middle of Te–Te layers (resulting in a clear vdW gap) which indicates a weak connection and confirms the weak stability of a vdW gap again. Besides, the bonds near the vdW gap (Ge_1_–Te_1_, Te_1_–Sb_1_–Te_2_, Te_2_–Ge_2_–Te_3_, Ge_3_–Te_3_) are stronger than the other bonds according to the value of ELF. These strong bonds provide the stability of vdW gap of the Kooi-mix model in the melting process at elevated temperature. Interestingly, the bonds connecting the inner Te atoms (Te_4_, Te_5_, and Te_6_) with the Sb1 atom is weaker than those of Ge_1_, Ge_2_, and Ge_3_ atoms. Hence, this is reason why the Sb atom is more likely to enter the vdW gap when annealing. Besides, it should be noted that the ELF of the atoms within the block is relatively symmetric, implying the occurrence of the resonant bond [18,21] in phase-change materials. Therefore, the resonant bond is probably a key to reveal the phase-change mechanism of GST-SL.

**Figure 5:**
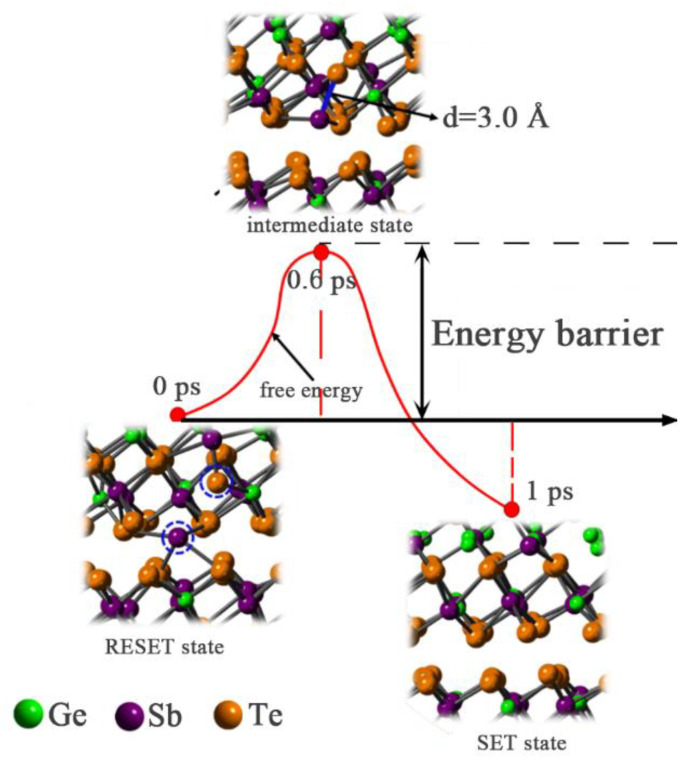
Schematic of the phase-change transition about GST-SL based on the Kooi-mix model.

We proposed a phase-change mechanism about the GST-SL based on the Kooi-mix model (shown in Figure 5). In this transition, the Kooi-mix model is considered as SET state. When heated by an optical or electrical pulse, the Sb atom enters the vdW gap and results in a vacancy in the Sb-rich layers. Meanwhile, the adjacent Te atom (marked by blue circle) moves toward the vacancy, resulting in the RESET state. When annealing the RESET structure of GST-SL at a proper temperature, the Sb atom in the vdW gap obtains the energy to exacerbate the thermal motion. Due to the long interaction of Sb-Te bonds (marked by a blue bond), the Sb atom is able to return to the previous position and complete the crystallization. Besides, we calculated the energy barriers (Ea) for the crystallization of GST-SL according to the free energy change during the crystallization. It was found that the Ea for this Sb atom transition was only 0.5 eV, far less than that in phase transition based on the Ge atom flipping (1.83–2.64 eV) [11,19,22]. In a summary, the Sb flipping transition is more acceptable than the Ge flipping.

**Figure 6:**
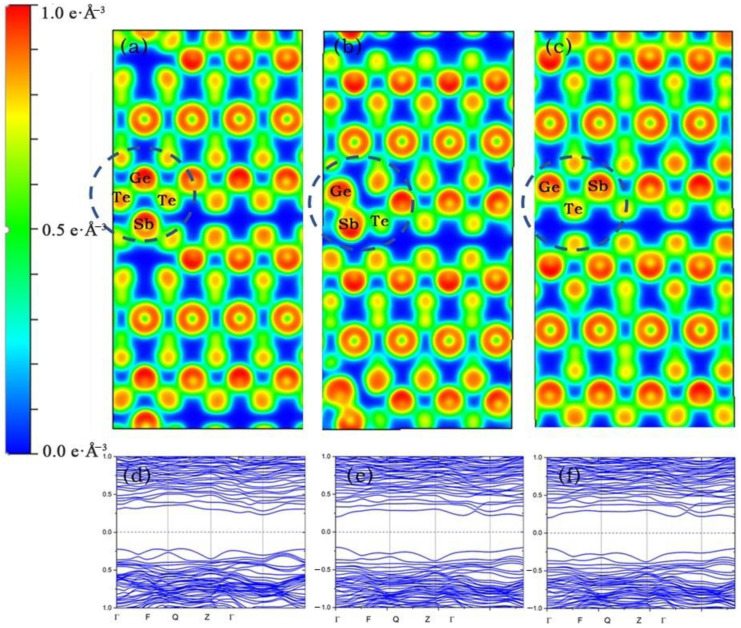
(**a**–**c**) represent the charge density of reset state, intermediate state, and set state, respectively, (**d**–**f**) represent the energy band calculation of reset state, intermediate state, and set state, respectively.

Finally, we performed energy band calculation and charge density analysis, GST-SL sampling was done using a 2 × 2 × 2 grid, all atoms were fully relaxed until the forces on each atom were smaller than 0.015 eV/Å, the Energy Convergence Standard was 10^−5^, energy cut off was 380 eV, convergence criterion of the force acting on each atom was −0.015, tetrahedron method with Blöchl corrections, K points used a 2 × 2 × 2 Gamma point grid. Figure 6 exhibits the energy bands of reset state and set state are the indirect band gap, the energy band of intermediate state is the direct band gap. With the process of crystallization, the values of the band gaps of GST-SL decreased gradually. According to electron localization function (ELF), according to color distribution, at reset state, there are almost no electrons in the Te–Te Van der Waals layer. The bond between the lowermost Sb atom and the Te atom above it is stronger than the bond connecting the Van der Waals layer, so Sb atoms are more likely to enter the Van der Waals layer; with the crystallization process the electron density between Sb atoms and the upper Te atoms increases, which proves that the Te–Sb bond appears. Under the pull of this bond, the Sb atoms jump and transform into a stable crystalline GST-SL state.

## 4. Discussion

The amorphization and crystallization processes of three GST-SL atomic models and GST models were simulated through first-principles molecular dynamics. In the simulation of the heating process, it was found that the layer structure of GST-SL makes its thermal stability better than that of GST. Additionally, from the free energy curve of GST-SL, it was found that its structure changes greatly at 1400 K. The amorphization simulations of three GST-SL models all showed the phenomenon of Sb entering and exiting a Te–Te paradigm layer before the layer structure was scattered, and the Kooi-mix model was the most stable structure in the GST-SL model. The Ferro-mix model cannot return to the original crystalline structure. In addition, the crystallization time of the GST model was greater than 150 ps. Therefore, the excellent crystalline properties of GST-SL come from its stable layer structure. Then, it was found in the crystallization simulation that both the crystallization time of the Kooi-mix and Petrov-mix models only take a few picoseconds. What is more, structural characteristics of the Koo-mix model and the reason why the Sb atom jumps into the paradigm layer was explained by analyzing the bond length distribution and the electronic local function. Based on the simulation results of amorphization and crystallization of GST-SL, this paper proposes a phase transition mechanism based on the Kooi-mix model, which is completely different from a phase transition mechanism of the highly recognized Ge atom jumping in the Te–Te paradigm layer [23,24]: after Sb atoms enter the Te–Te paradigm layer of GST-SL, the Te atoms above it face Te–Te layer collapses in the direction. Sb atoms are captured by the collapsed Te atoms when they vibrate up and down under the action of the crystallization temperature to form a Te–Sb bond. Finally, the Sb atoms jumped back to their original positions under the pull of this bond. In addition, the phase-change activation energy for this crystallization process only requires 0.5 eV, which is far lower than that used in the Ge atom jumping mechanism. This paper proposes a Sb atom hopping mechanism, which has reference significance for the research on the phase change mechanism of GST-SL and the optimization of phase change memory.

## 5. Conclusions

In this work, in order to explore the phase transition mechanism of GST-SL, we used AIMD simulation to study three phase transition characteristics of GST-SL models based on Ge/Sb mixture. The results revealed that in the melting process, the Kooi-mix model had a longer melting time than Petrov-mix and Ferro-mix models. (30 ps vs. 8 ps), compared to the other two models, the Ge–Te bonds in the Kooi-mix model were stronger. Therefore, Kooi-mix model has the best structural stability compared with the Petrov-mix and Ferro-mix models, the Kooi-mix model is more in line with the phase transition mechanism of GST-SL. The structure transition characteristics of GST-SL are explained by the key distribution and electronic positioning function. The phase transition caused by the jumping of Sb atoms required only 0.5 eV of phase change activation energy, which was much lower than the activation energy of the Ge atom jumping mechanism, and Sb atoms more easily jumped into the paradigm layer than Ge atoms, and this was also verified by electron density calculation and energy band calculation. In light of these results, we propose a new a phase transition mechanism for GST-SL based on Sb flipping. The current findings help to study the phase transition mechanism of GST-SL and optimize the performance of PCM.

## Data Availability

The data presented in this study are available in supplementary materials.

## Supplementary Materials

The following are available online at https://www.mdpi.com/1996-1944/14/2/360/s1, Figure S1: Modeling process of the GST-SL atomic structure (green spheres are Ge, purple spheres are Sb, and orange spheres are Te); (a) Kooi, (b) Ferro, (c) Petrov, (d) Kooi-mix, (e) Ferro-mix, (f) Petrov-mix. The crystal lattice parameters (a = 4.12 Å, c = 17.2 Å) and space group (R$\bar{3}$m) refer to the previous study, Figure S2: The curves of time-temperature and time-free energy about Kooi-mix model in the heating processes.
