# Supplementary material for: A Phase-Change Mechanism of GST-SL Based Superlattices upon Sb Flipping
Source: Materials (Basel). 2021 Jan 13;14(2):360. doi: 10.3390/ma14020360 (PMC7828381; doi:10.3390/ma14020360)
Supplement: Supplementary file 1 [file materials-14-00360-s001.pdf]

materials

Supplementary Material

A Phase-Change Mechanism of GST-SL Based Superlattices upon Sb Flipping

Teng Sun [1,2,3], Furong Liu [1,2,3,*], Jicheng Guo [1,2,3], Gang Han [1,2,3] and Yongzhi Zhang [1,2,3]

[1] Key Laboratory of Trans-scale Laser Manufacturing Technology, Beijing University of Technology, Ministry of Education,100 Ping Leyuan, Chaoyang District, Beijing 100124, China; sunny@emails.bjut.edu.cn (T.S.); smart.guo@hotmail.com (J.G.); yeahhanpei@163.com (G.H.); ZhangYZ@bjut.edu.cn (Y.Z.)
[2] Beijing Engineering Research Center of Laser Technology, Beijing University of Technology,100 Ping Leyuan, Chaoyang District, Beijing 100124, China
[3] Institute of Laser Engineering, Faculty of Materials and Manufacturing, Beijing University of Technology, 100 Ping Leyuan, Chaoyang District，Beijing 100124, China
* Correspondance: Liufr@bjut.edu.cn; Tel.: +86-010-67396559; Fax: +86-010-67392773

Citation: Sun, T.; Liu, F.; Guo, J.; Han, G.; Zhang, Y. A Phase-Change Mechanism of GST-SL Based Superlattices upon Sb Flipping. *Materials* **2021**, *14*, x. https://doi.org/10.3390/xxxxx

Received: 10 November 2020
Accepted: 9 January 2021
Published: 13 January 2021

Publisher's Note: MDPI stays neutral with regard to jurisdictional claims in published maps and institutional affiliations.

Copyright: © 2021 by the authors. Licensee MDPI, Basel, Switzerland. This article is an open access article distributed under the terms and conditions of the Creative Commons Attribution (CC BY) license (http://creativecommons.org/licenses/by/4.0/).

1. Supplementary Information

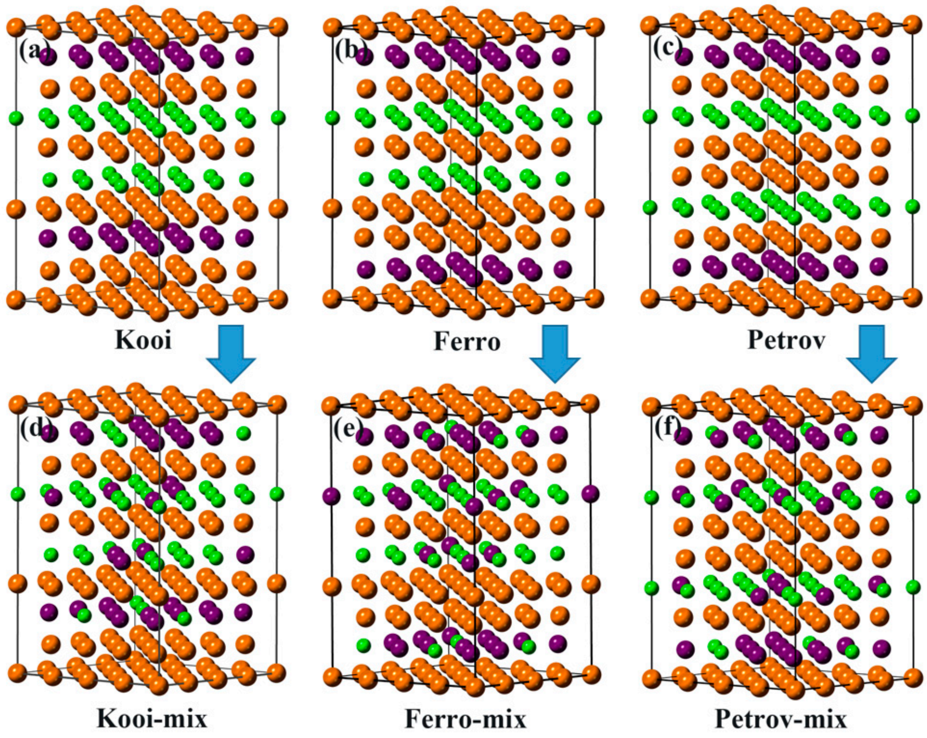

Figure S1. Modeling process of the GST-SL atomic structure (green spheres are Ge, purple spheres are Sb, and orange spheres are Te); (**a**) Kooi, (**b**) Ferro, (**c**) Petrov, (**d**) Kooi-mix, (**e**) Ferro-mix, (**f**) Petrov-mix. The crystal lattice parameters (a = 4.12 Å, c = 17.2 Å) and space group (R$\bar{3}$m) refer to the previous study [1].

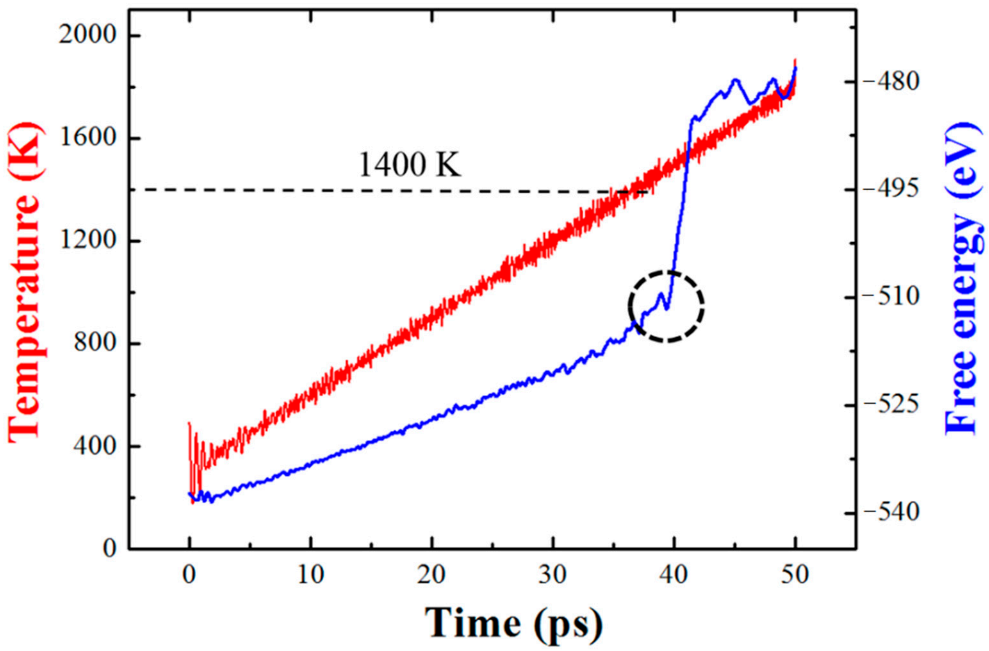

Figure S2. The curves of time–temperature and time–free energy about Kooi-mix model in the heating processes.

References

1. Tominaga, J.; Kolobov, A.; Fons, P.; Nakano, T.; Murakami, S., Ferroelectric Order Control of the Dirac-Semimetal Phase in GeTe-Sb2Te3 Superlattices. *Adv. Mater. Interfaces* **2014**, *1*, 1300027.
